# Supplementary material for: Expanded catalog of microbial genes and metagenome-assembled genomes from the pig gut microbiome
Source: Nat Commun. 2021 Feb 17;12:1106. doi: 10.1038/s41467-021-21295-0 (PMC7889623; doi:10.1038/s41467-021-21295-0)
Supplement: Supplementary file 1 — Supplementary Information [file 41467_2021_21295_MOESM1_ESM.pdf]

SUPPLEMENTARY INFORMATION

Expanded catalogue of microbial genes and metagenome-assembled genomes from the pig gut microbiome

Congying Chen[†*], Yunyan Zhou[†], Hao Fu, Xinwei Xiong, Shaoming Fang, Hui Jiang, Jinyuan Wu, Hui Yang, Jun Gao, Lusheng Huang[*]

State Key Laboratory of Pig Genetic Improvement and Production Technology, Jiangxi Agricultural University, 330045, Nanchang, China

[†]These authors contribute equally: Congying Chen, Yunyan Zhou

[*]These authors jointly supervised this work: Congying Chen, Lusheng Huang

Running title: Gene catalogue and metagenome-assembled genomes of pig gut microbiome

***Correspondence authors**

Congying Chen, Lusheng Huang

Phone: 0086-791-83813080; Fax: 0086-791-83900189

E-mail: chcy75@hotmail.com (Congying Chen); Lushenghuang@hotmail.com (Lusheng Huang)

Supplementary Figures

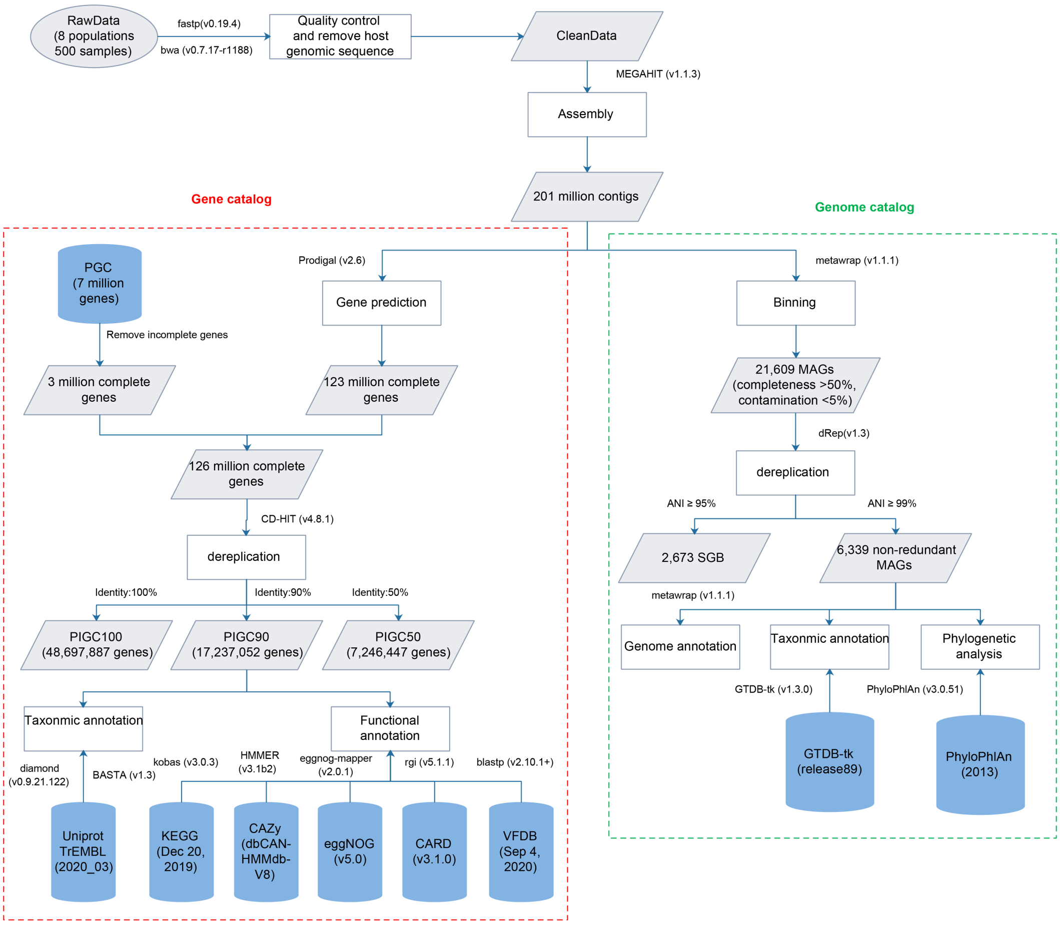

Supplementary Figure 1 | Methodology overview for the construction of reference gene catalog and metagenome-assembled genomes. There are two main parts in the pipeline: the construction of the gene catalog (red dotted box) and metagenome-assembled genomes (green dotted box). In this flowchart, the grey ellipse represents raw metagenomic sequencing data, rectangles represent the data processing, grey rhombuses represent input or output files in each step, blue cylinders represent the public database, and the text beside the arrows on the flow chart indicates the software or the thresholds. The microbial genes in the PGC reported previously were integrated in the PIGC (pig integrated gene catalog).

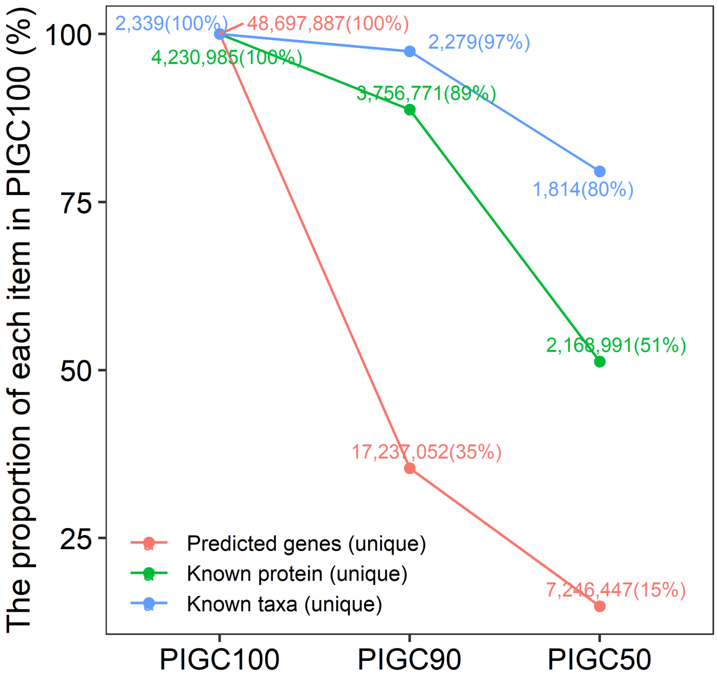

Supplementary Figure 2 | The numbers (percentages) of predicted genes, and known protein and taxa for three gene catalogs. The annotation of known protein and taxa was performed by aligning protein sequence to the Uniprot TrEMBL. The value next to the dot indicates the number of each item, and the values in parentheses indicate the proportion of each item in the total number of genes in the PIGC100.

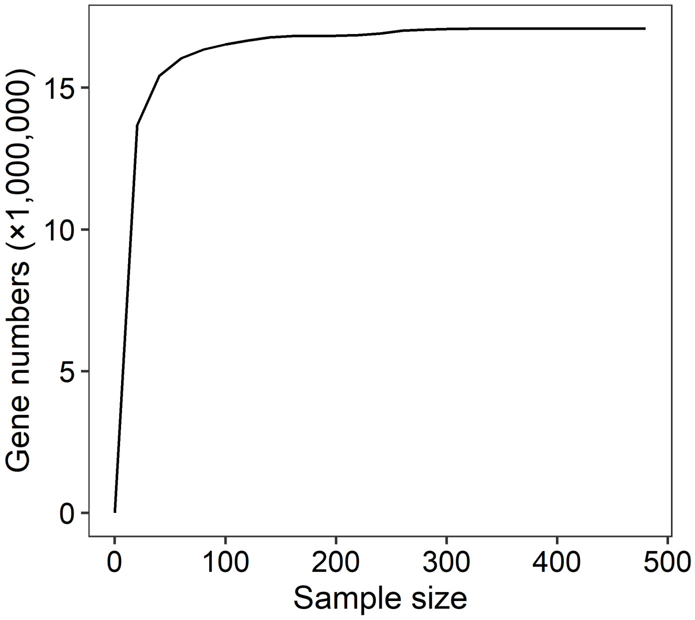

Supplementary Figure 3 | Rarefaction curve of detected gene numbers from all 500 samples. Rarefaction analysis was performed by random sampling 100 times without replacement for a given number of sample size and calculated the total number of nonredundant genes in each sampling. The sample size was increased from 20 to 500 at the rate of 20 samples per step. The averages of 100 times sampling for each sample size were plotted.

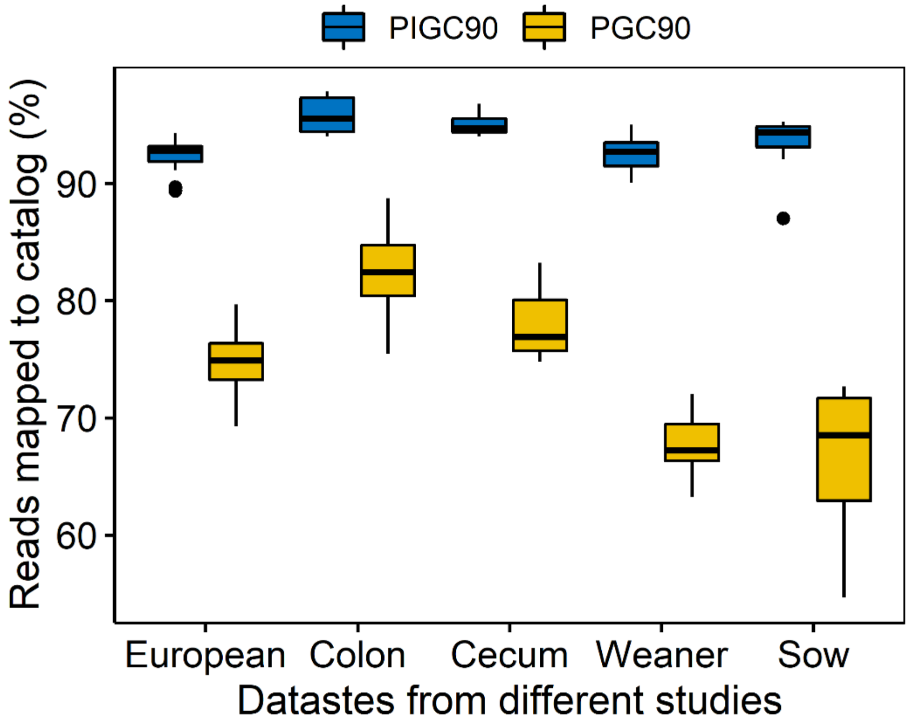

Supplementary Figure. 4 | Percentages of the sequence reads that could be mapped to the PIGC90 and PGC90 using metagenomic sequencing data from five unrelated studies. To evaluate the representation of the PIGC, 76 metagenomic sequencing data from five different sample sources including feces of European pigs (n=20), feces of weaner (n=20) and sow (n=20), colon luminal contents (n=8), cecum luminal contents (n=8), were downloaded from four public datasets and mapped to the PIGC90 and PGC90. For the PIGC90, an average of 93% (ranging from 87.03% to 97.83%) of mapped reads was obtained, and for the PGC90, the average percentage of the mapped reads was 72% (ranging from 54.65% to 88.72%). In the boxplot, the middle line is the median, the boxes are the lower and upper quartiles, the whiskers are the minima and maxima, points laying outside the whiskers of the boxplot represents the outlier.

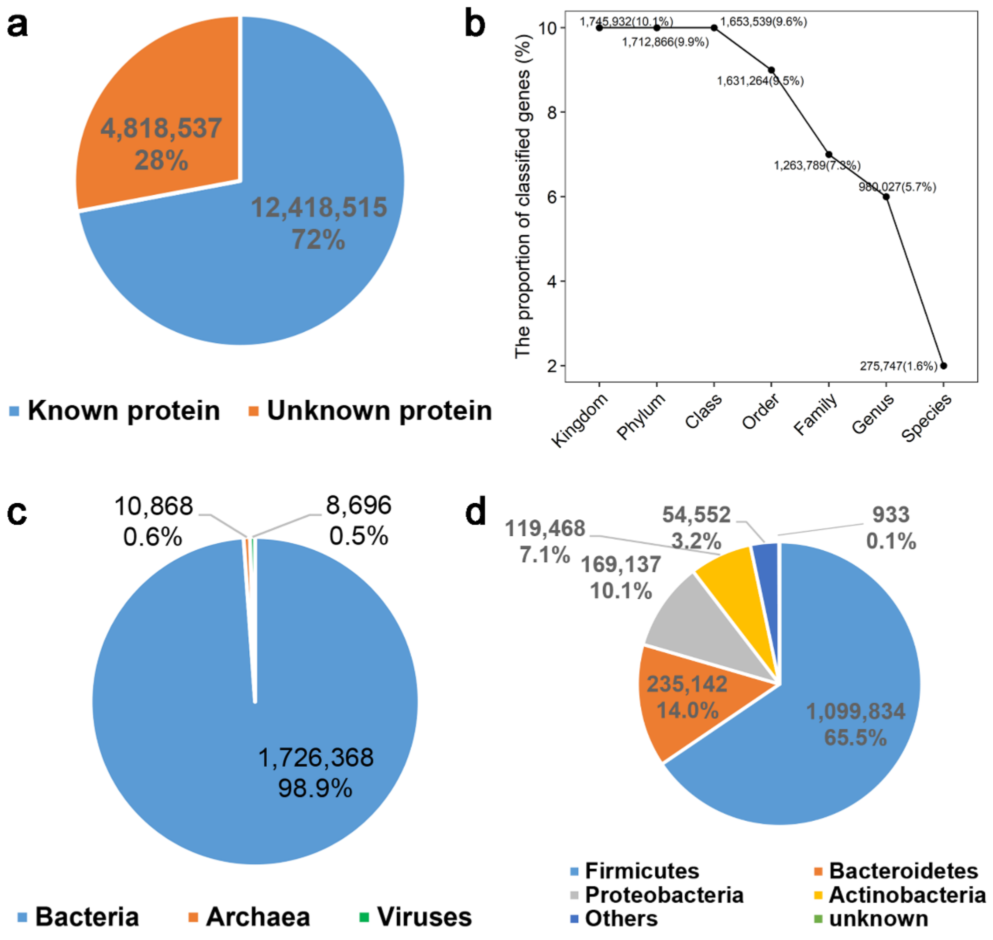

Supplementary Figure 5 | Annotation of genes in the PIGC90. (a) The number and percentage of the known and unknown proteins in the PIGC90. A protein was defined as the known protein if its protein sequence could be aligned in Uniprot TrEMBL database. (b) The number (percentage) of genes that could be classified to each taxonomic level in the PIGC90. (c) The number and percentage of genes classified to bacteria, archaea and viruses. (d) The number and percentage of genes classified to different phyla of bacteria.

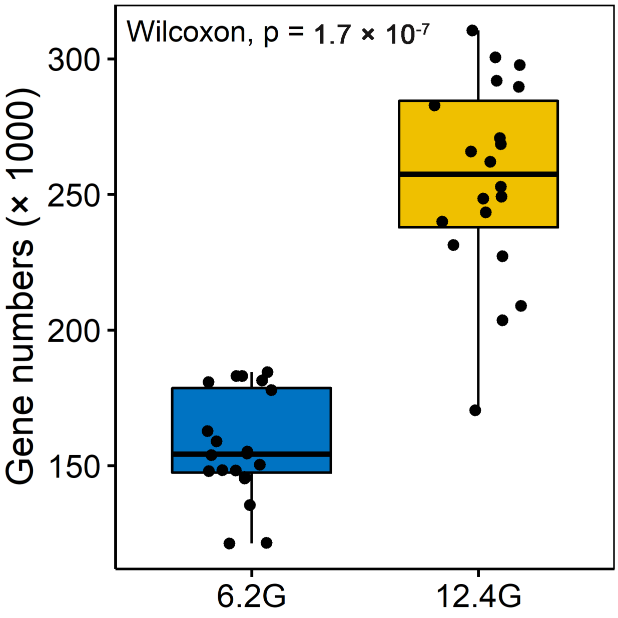

Supplementary Figure 6 | Comparison of predicted gene number between high and low sequencing depth. Significantly more predicted genes were identified in the samples with high sequencing depth (6.2 Gb *vs*. 12.4Gb, n = 20). The comparison was performed with the two-tailed Wilcoxon test was used. Boxplots show median, 25th and 75th percentile, the whiskers indicate the minima and maxima, and the points laying outside the whiskers of boxplots represent the outliers.

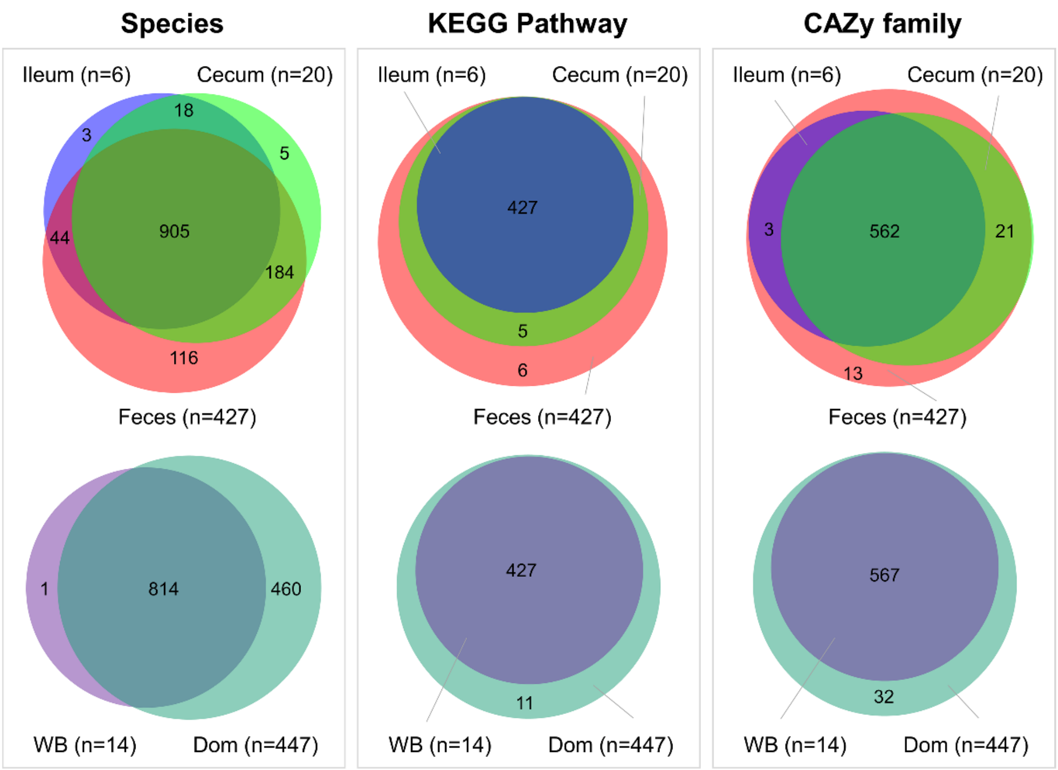

Supplementary Figure 7 | The specific and shared bacteria species, KEGG pathways and CAZymes among different gut locations, and between wild boars and domestic pigs. The samples used for the comparisons, including ileum lumen, cecum lumen and feces samples were from wild boars and adult domestic pigs.

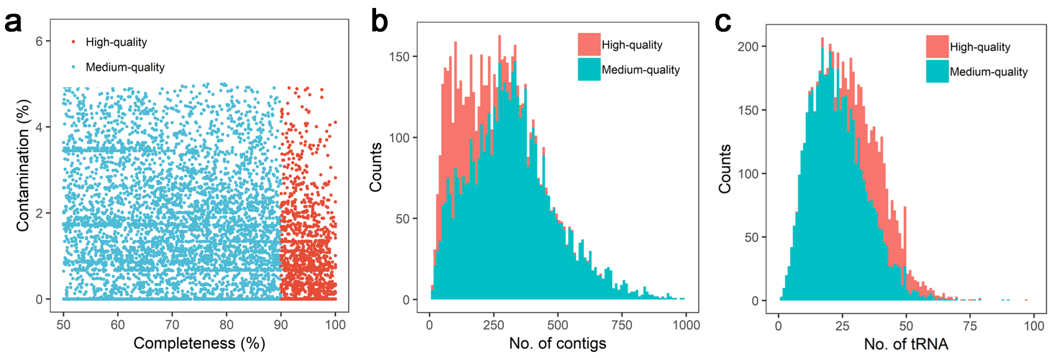

Supplementary Figure 8 | The genomic feature of 6,339 metagenome-assembled genomes (MAGs). (a) The completeness and contamination of MAGs. the 6,339 non-redundant MAGs were divided into medium-quality MAGs (more than 50% completeness and less than 5% contamination) and high-quality MAGs ((more than 90% completeness and less than 5% contamination). (b) The distribution of contig numbers for medium-quality MAGs and high-quality MAGs. (c) The distribution of tRNA numbers for medium-quality MAGs and high-quality MAGs.

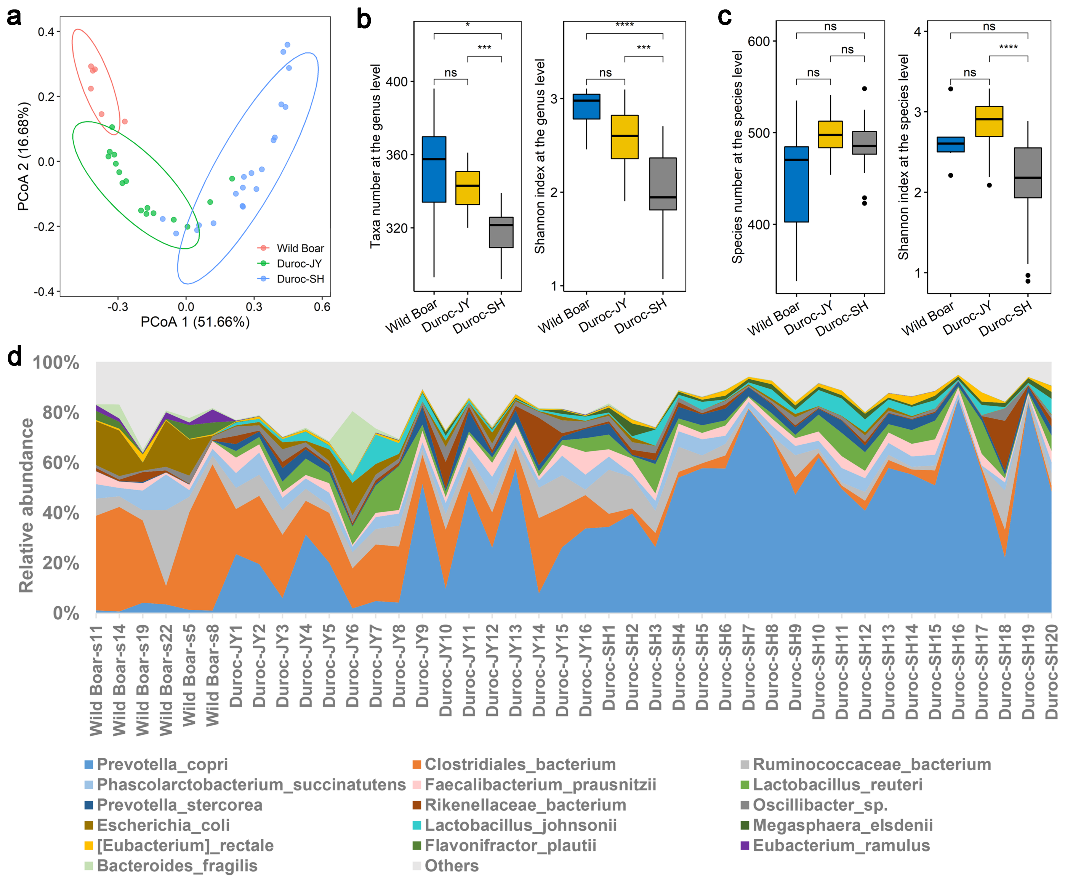

Supplementary Figure 9 | Comparison of the composition and diversity of gut microbiome between wild boars and Duroc pigs. (a) Distinct gut microbial compositions between wild boars and Duroc pigs by PCoA analysis. (b) Comparison of the α-divereity of gut microbiome at the genus level among wild boars (n=6), Duroc-JY (n=16) and Duroc-SH (n=20). * $P < 0.05$, ** $P < 0.01$, *** $P < 0.001$, **** $P < 0.0001$, two-tailed Wilcoxon test was used. Boxplots show median, 25th and 75th percentile, the whiskers indicate the minima and maxima, and the points laying outside the whiskers of boxplots represent the outliers. (c) Comparison of the α-divereity of gut microbiome at the species level among wild boars (n=6), Duroc-JY (n=16) and Duroc-SH (n=20). * $P < 0.05$, ** $P < 0.01$, *** $P < 0.001$, **** $P < 0.0001$, two-tailed Wilcoxon test. (d) Distribution of the relative abundances of the 20 most abundant

species among wild boars, Duroc-JY and Durco-SH pigs. The X-axis shows the sample ID, and the Y-axis indicates the relative abundance of bacterial species. Ten most abundant bacterial species from each population were included.

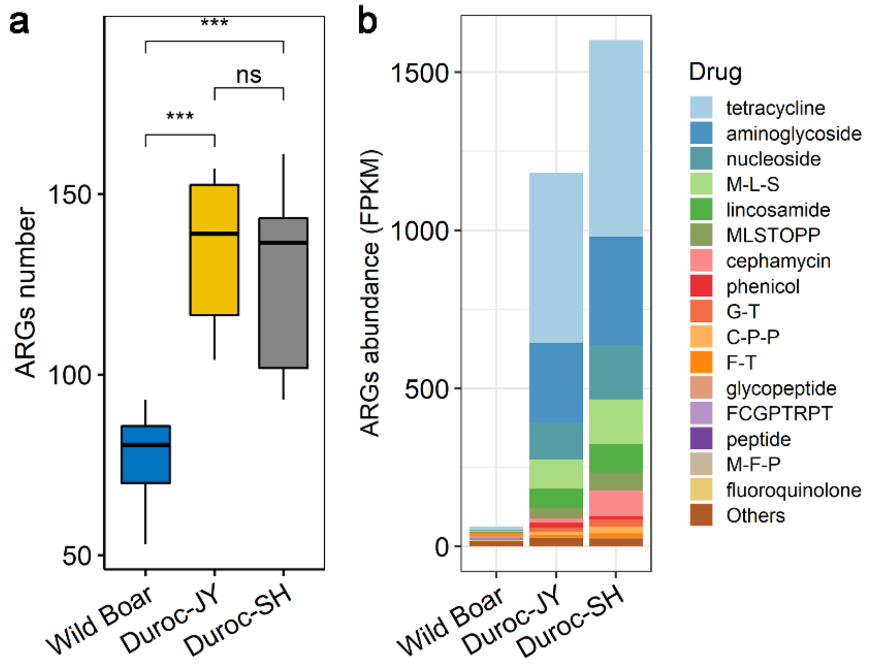

Supplementary Figure 10 | Comparison of the numbers and abundances of antibiotic resistant genes (ARGs) between wild boars and two Duroc pig populations. (a) Comparison of the numbers of ARGs. Significantly different numbers of ARGs were observed between wild boars (n=6) and Duroc pigs (Duroc-JY: n=16, Duroc-SH: n=20, two-tailed Wilcoxon test was used), *FDR* corrected *P* values are as follows: wild boars *vs*. Duroc-JY, $P = 6.9 \times 10^{-4}$; wild boars *vs*. Duroc-SH, $P = 6.9 \times 10^{-4}$, Duroc-JY *vs*. Duroc-SH, $P = 0.24$. * $P < 0.05$, ** $P < 0.01$, *** $P < 0.001$, **** $P < 0.0001$. Boxplots show median, 25th and 75th percentile, the whiskers indicate the minima and maxima, and the points laying outside the whiskers of boxplots represent the outliers. (b) The abundance of each class of ARGs in wild boars, Duroc-JY and

Duroc-SH. The bars were colored by the resistance classes of ARGs to antibiotics. The resistance classes of ARGs and the abbreviations of antibiotics were listed in Supplementary Table 4.

Supplementary Tables

Supplementary Table 1. Summary of the samples used in this study.

Population	Sample type	Age (day)	Sex	Farm location	Sample size	# Raw Reads (million)	# Raw Base pairs (Gb)	# Clean Reads (million)	# Clean Base pairs (Gb)
F6 pigs of Mosaic population	feces	240	Male:127 Female: 174	Nanchang	301	31.28	4,692	27.09	4,062
	feces	25	Male:3 Female:7	Nanchang	10				
	cecum	240	Male:4 Female:6	Nanchang	10				
	ileum	240	Male:1 Female:3	Nanchang	4				
Duroc	feces	160	Male:10 Female: 10	Shahu	20	1.19	149	1.18	148
	feces	160	Male: 8 Female: 8	Jiangying	16	1.13	169	1.09	163
Wild Boar	feces	adult	unknown	NA	6	1.23	184	1.02	152
	cecum	adult	unknown	NA	8				
Tibetan	feces	210	Male:12 Female:9	Kangding	21	2.32	348	1.82	273
	feces	210	Male:3 Female:3	Nanchang	6				
Large White	feces	30	Male:6 Female:13	Liangyeshan	19	1.49	224	1.11	167
	feces	30	Male:6 Female:4	Liangyeshan	10	1.67	175	1.01	151
Laiwu	cecum	300	pool	Nanchang	2	0.62	62	0.49	49
	ileum	300	pool	Nanchang	2				
	jejunum	300	pool	Nanchang	2				
Berkshire × Licha line	feces	342~951	female	Dingnan	49	4.41	661	3.77	565
Licha	feces	349~758	female	Dingnan	14				
In total					500	44.84	6,665	38.58	5,730

Supplementary Table 2. Number and percentage of the genes in the PIGC catalog annotated to the items of each database.

**Item**	**Number**	**Percentage (%)**
Non-redundant genes	17,237,052	100.00%
Genes blasted to Uniprot TrEMBL database	12,418,515	72.05%
Genes annotated to KEGG Orthologs/Ortholog Number	2,853,603/6,363	16.56%
Genes annotated to KEGG pathways/Pathway Number	2,853,603/438	16.56%
Genes annotated to eggNOG/Ortholog Number	10,606,969/81406	61.54%
Genes annotated to CAZy/CAZy Familys	2,045,161/599	11.86%
Genes annotated to CARD/ARO Number	916/333	0.01%
Genes annotated to VFDB/VF Number	2,184,919/1,633	12.68%

Supplementary Table 3. The top 10 function capacities of pig gut microbiome in abundance based on CAZy, eggNOG and KEGG pathway.

Database	Name	Description	Average abundance (FPKM)
CAZy family	GT2	GlycosylTransferase Family 2	4256.45
	GT4	GlycosylTransferase Family 4	2653.63
	GT41	GlycosylTransferase Family 41	1771.17
	CE9	Carbohydrate Esterase Family 9	1367.15
	CBM50	Carbohydrate-Binding Module Family 50	1296.44
	CE1	Carbohydrate Esterase Family 1	1172.72
	AA3	Auxiliary Activity Family 3	1083.42
	GH2	Glycoside Hydrolase Family 2	1057.16
	CE4	Carbohydrate Esterase Family 4	1051.16
	GH109	Glycoside Hydrolase Family 109	914.92
eggNOG Ortholog	COG1961	recombinase activity	4510.05
	COG1396	sequence-specific DNA binding	3649.94
	COG0582	DNA integration	3495.33
	COG2801	transposition	3219.07
	COG1132	ATPase activity, coupled to transmembrane movement of substances	3165.73
	COG0438	transferase activity, transferring glycosyl groups	3131.65
	COG0745	phosphorelay signal transduction system	2993.05
	COG4974	DNA integration	2917.85
	COG0534	drug transmembrane transporter activity	2806.94
	COG0456	peptide alpha-N-acetyltransferase activity	2796.18
KEGG pathway	ko02010	ABC transporters	20483.46
	ko03010	Ribosome	19802.97
	ko02020	Two-component system	15608.34
	ko00230	Purine metabolism	15501.07
	ko00240	Pyrimidine metabolism	12468.48
	ko00970	Aminoacyl-tRNA biosynthesis	12468.43
	ko02024	Quorum sensing	12284.7
	ko03440	Homologous recombination	11610.95
	ko00520	Amino sugar and nucleotide sugar metabolism	11608.5
	ko00010	Glycolysis / Gluconeogenesis	10522.12

Supplementary Table 4. Comparison of the abundance of antibiotic resistance genes between wild boars and Duroc pigs.

Resistance classes	Abbreviation of antibiotics	Duroc-JY (FPKM)	Duroc-SH (FPKM)	Wild Boar (FPKM)
aminocoumarin antibiotic	aminocoumarin	0.397	0.228	1.430
aminoglycoside antibiotic	aminoglycoside	252.755	342.598	2.268
aminoglycoside antibiotic; aminocoumarin antibiotic	A-A	0.490	0.241	1.502
carbapenem	carbapenem	0	0	0.012
carbapenem; cephalosporin; penam	C-C-P	0.675	0.364	0
cephalosporin	cephalosporin	0.198	0.020	0.071
cephalosporin; cephamycin	C-C	0.006	0	0
cephalosporin; cephamycin; penam	C-C1-P	0.101	0.053	0.368
cephalosporin; penam	C-P	2.777	1.855	1.444
cephalosporin; penam; penem	C-P-P	9.439	20.139	0
cephamycin	cephamycin	14.882	81.411	0.143
diaminopyrimidine antibiotic	diaminopyrimidine	0.383	0.869	0
elfamycin antibiotic	elfamycin	0.439	0.235	1.061
fluoroquinolone antibiotic	fluoroquinolone	0.843	0.549	2.310
fluoroquinolone antibiotic; aminoglycoside antibiotic; tetracycline antibiotic; rifamycin antibiotic	F-A-T-R	0	0	0.005
fluoroquinolone antibiotic; cephalosporin; cephamycin; penam	F-C-C-P	0.463	0.295	2.026
fluoroquinolone antibiotic; cephalosporin; glycylcycline; cephamycin; penam; tetracycline antibiotic; rifamycin antibiotic; phenicol antibiotic; triclosan	FCGCPTRPT	0.182	0.095	0.586
fluoroquinolone antibiotic; cephalosporin; glycylcycline; penam; tetracycline antibiotic; acridine dye; rifamycin antibiotic; phenicol antibiotic; triclosan	FCGPTARPT	0	0	0.010
fluoroquinolone antibiotic; cephalosporin; glycylcycline; penam; tetracycline antibiotic; rifamycin antibiotic; phenicol antibiotic; triclosan	FCGPTRPT	0.711	0.411	2.727
fluoroquinolone antibiotic; glycylcycline; tetracycline antibiotic; diaminopyrimidine antibiotic; nitrofuran antibiotic	F-G-T-D-N	0.033	0.008	0.017
fluoroquinolone antibiotic; lincosamide antibiotic; nucleoside antibiotic; acridine dye; phenicol antibiotic	F-L-N-A-P	0.157	0.103	0.769
fluoroquinolone antibiotic; monobactam; carbapenem; cephalosporin; glycylcycline; cephamycin; penam; tetracycline antibiotic; rifamycin antibiotic; phenicol antibiotic; triclosan; penem	FMCCGCPTRPTP	0.207	0.135	0.889
fluoroquinolone antibiotic; tetracycline antibiotic	F-T	7.088	12.278	9.940
fosfomycin	fosfomycin	0.228	0.107	1.001
glycopeptide antibiotic	glycopeptide	1.455	2.101	6.652
glycylcycline; tetracycline antibiotic	G-T	13.325	22.987	0.004
lincosamide antibiotic	lincosamide	59.508	90.280	0.578

lincosamide antibiotic; streptogramin antibiotic; oxazolidinone antibiotic; phenicol antibiotic; pleuromutilin antibiotic	L-S-O-P-P	0.010	0.004	0.004
lincosamide antibiotic; streptogramin antibiotic; phenicol antibiotic; pleuromutilin antibiotic	L-S-P-P	5.876	0.002	0
macrolide antibiotic	macrolide	5.565	11.755	0.573
macrolide antibiotic; aminoglycoside antibiotic; cephalosporin; tetracycline antibiotic; peptide antibiotic; rifamycin antibiotic	MACTPR	0.248	0.079	0.556
macrolide antibiotic; fluoroquinolone antibiotic	MF	0	0	0.003
macrolide antibiotic; fluoroquinolone antibiotic; aminoglycoside antibiotic; carbapenem; cephalosporin; glycylcycline; cephamycin; penam; tetracycline antibiotic; peptide antibiotic; aminocoumarin antibiotic; rifamycin antibiotic; phenicol antibiotic; triclosan; penem	MFACCGPTPARPRP	0.137	0.108	0.528
macrolide antibiotic; fluoroquinolone antibiotic; aminoglycoside antibiotic; carbapenem; cephalosporin; penam; peptide antibiotic; penem	MFACCPPP	0	0	0.017
macrolide antibiotic; fluoroquinolone antibiotic; aminoglycoside antibiotic; cephalosporin	M-F-A-C	0.001	0.005	0.024
macrolide antibiotic; fluoroquinolone antibiotic; cephalosporin; cephamycin; penam; tetracycline antibiotic	MFCCPT	0.096	0.029	0.361
macrolide antibiotic; fluoroquinolone antibiotic; cephalosporin; fusidic acid	M-F-C-F	0	0.002	0
macrolide antibiotic; fluoroquinolone antibiotic; penam	M-F-P	0.717	0.433	2.359
macrolide antibiotic; fluoroquinolone antibiotic; penam; tetracycline antibiotic	M-F-P-T	0.286	0.197	1.016
macrolide antibiotic; fluoroquinolone antibiotic; rifamycin antibiotic	MFR	0	0	0.003
macrolide antibiotic; lincosamide antibiotic	M-L	0.004	0	0
macrolide antibiotic; lincosamide antibiotic; streptogramin antibiotic	M-L-S	91.711	141.406	4.465
macrolide antibiotic; lincosamide antibiotic; streptogramin antibiotic; tetracycline antibiotic; oxazolidinone antibiotic; phenicol antibiotic; pleuromutilin antibiotic	MLSTOPP	33.253	56.388	2.208
monobactam; cephalosporin; penam; penem	M-C-P-P	0.149	0.251	0.054
nitroimidazole antibiotic	nitroimidazole	0.102	0.053	0.352
nucleoside antibiotic	nucleoside	116.879	171.009	0.351
nucleoside antibiotic; acridine dye	N-A	0.506	0.313	2.022
para-aminosalicylic acid	P	0.001	0.004	0.008
penam	penam	0.332	0.070	0.005
peptide antibiotic	peptide	0.741	0.471	2.393
phenicol antibiotic	phenicol	14.403	11.033	0.010
rifamycin antibiotic	rifamycin	0.001	0.007	0.279
streptogramin antibiotic	streptogramin	4.683	7.022	0
sulfonamide antibiotic	sulfonamide	1.182	1.210	0.022
tetracycline antibiotic	tetracycline	537.735	622.235	9.631
tetracycline antibiotic; benzalkonium chloride; rhodamine	T-B-R	0.148	0.066	0.430
