# Supplementary material for: Expanded catalog of microbial genes and metagenome-assembled genomes from the pig gut microbiome
Source: Nat Commun. 2021 Feb 17;12:1106. doi: 10.1038/s41467-021-21295-0 (PMC7889623; doi:10.1038/s41467-021-21295-0)
Supplement: Supplementary file 3 — Description of Additional Supplementary Files [file 41467_2021_21295_MOESM3_ESM.docx]

**Description of Additional Supplementary Files**

**Supplementary Data 1**

Statistical summary of 6,339 metagenome assembled genomes. This supplementary data provides completeness, contamination, contig number, tRNA number, quality classification, genomic size (bp), N50 value, cluster ID and quality score in dRep, frequency in 500 tested samples, and the annotation with GTDB database for all 6,339 metagenome assembled genomes.

**Supplementary Data 2**

The description of species-level genome bins (SGBs). The column 4 indicates whether the SGBs are unknown SGBs (uSGB), the column 5 indicates the number of MAGs having reference genome in SGBs, the column 6 shows the number of reconstructed genomes in the SGBs, the columns 7 and 8 indicate the ID and name of representative MAG, and the column 9 shows the representative taxa of SGBs.

**Supplementary Data 3**

The species enriched in the gut microbiome of Wild boars and Duroc pigs. This supplementary data lists all information of bacterial species showing differential abundances between Wild boars and Duroc-SH, and between Wild boars and Duroc-JY, including abundances (mean ± sd, FPKM) and significant P values.

**Supplementary Data 4**

The MAGs enriched in the gut microbiome of Wild boars and Duroc pigs. This supplementary data lists all information of MAGs showing differential abundances between Wild boars and Duroc-Shahu, and between Wild boars and Duroc-Jiangying, including abundances (mean ± sd, FPKM), significant P values, annotation of MAGs based on GTDB database, and the SGB ID containing differential MAGs.

**Supplementary Data 5**

The KEGG pathway enriched in the gut microbiome of Wild boars and Duroc pigs. This supplementary data lists all KEGG pathways showing differential abundances between Wild boars and Duroc pigs, including abundances (mean ± sd, FPKM) and significant P values.
